# Supplementary material for: BIGDML—Towards accurate quantum machine learning force fields for materials
Source: Nat Commun. 2022 Jun 29;13:3733. doi: 10.1038/s41467-022-31093-x (PMC9243122; doi:10.1038/s41467-022-31093-x)
Supplement: Supplementary file 1 — Supplementary Information [file 41467_2022_31093_MOESM1_ESM.pdf]

SUPPLEMENTARY INFORMATION

"BIGDML – Towards Accurate Quantum Machine Learning Force Fields for Materials"

H.E. Sauceda *et al.*

SUPPLEMENTARY FIGURES

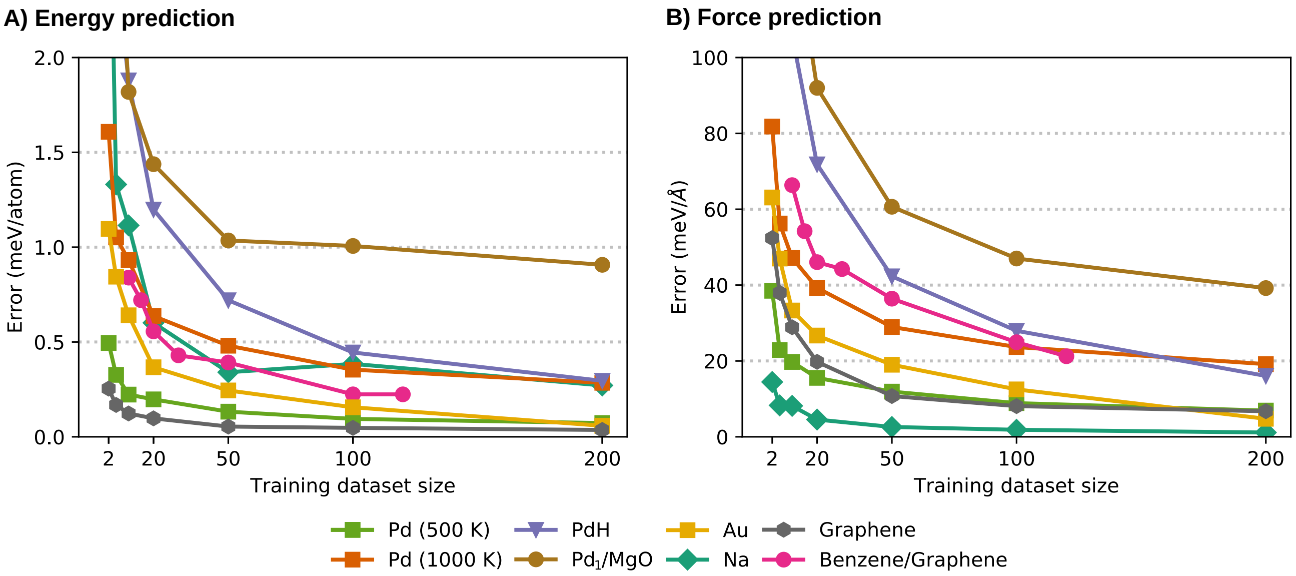

Supplementary Figure 1. Root-mean-square error (RMSE) learning curves for different materials. 3D bulk materials: Pd(FCC), Na(BCC), and Au(FCC). 2D material: Graphene. Interstitial in materials: H in a supercell of Pd. Chemisorption of atom at a surface: Single Pd atom adsorbed on a MgO (100) surface. Van der Waals interactions: Benzene molecule adsorbed on graphene.

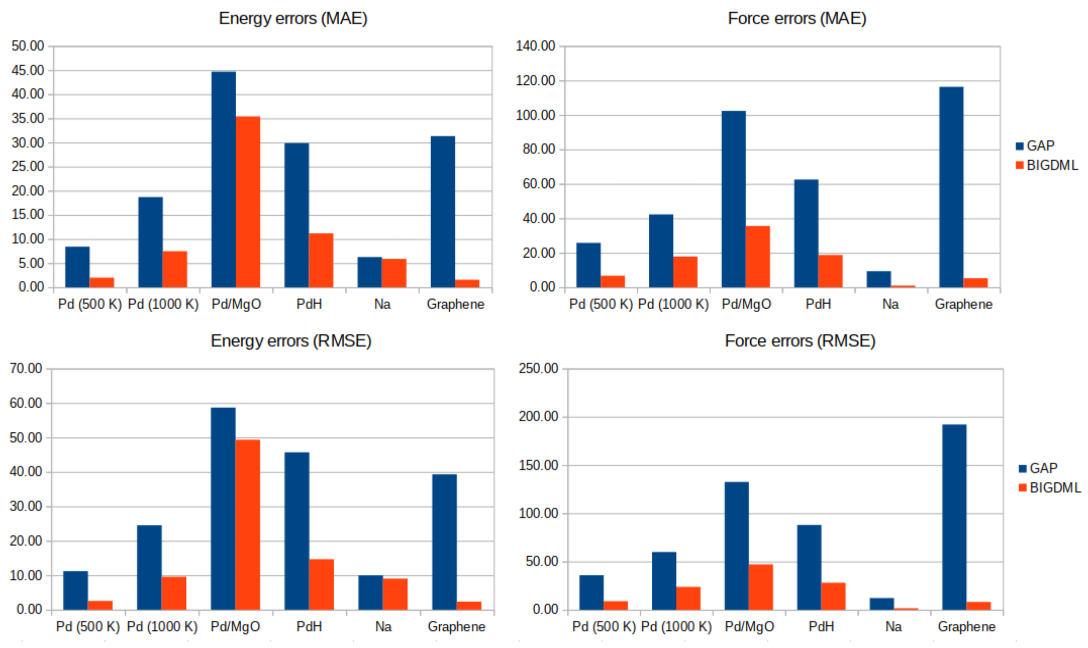

Supplementary Figure 2. Comparison between BIGDML and GAP/SOAP generalization errors for different systems trained on 100 samples.

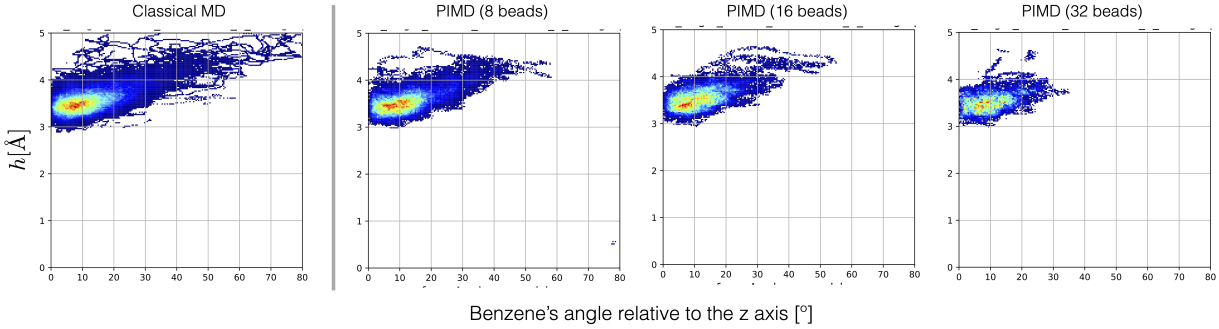

Supplementary Figure 3. Incremental inclusion the nuclear quantum effects on Benzene/graphene by increasing the number of beads in the path integral molecular dynamics simulation at 300 K. The two main degrees of freedom: (1) The angle between the normal vector defined by the benzene ring $\hat{\mathbf{n}}$ and the normal to the graphene plane ($\hat{\mathbf{z}}$). (2) The relative distance between the benzene center of mass and the graphene, h.

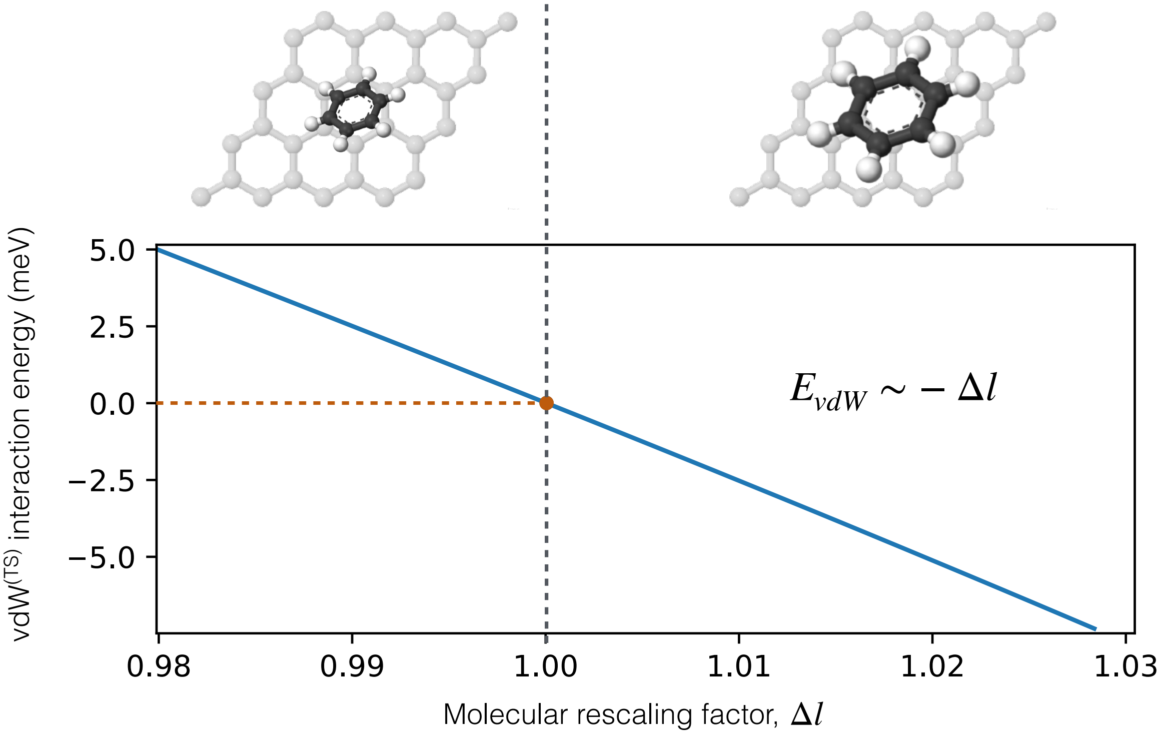

Supplementary Figure 4. Implications of the effective molecular volume on van der Waals forces. Starting from the parallel local minima of the benzene/graphene, the coordinates were scaled by Δl to change the molecular volume keeping h fixed. The calculations were done using DFT using the PBE exchage-correlation functional and the Tkatchenko-Scheffler (TS) pairwise method for van der Waals interactions. [68, 91]

SUPPLEMENTARY NOTES

I. VALIDATION BEYOND SUMMARY ERROR: PALLADIUM FCC AT DIFFERENT TEMPERATURES

As an additional validation of the BIGDML model beyond conventional machine learning summary errors, here, we consider the case of a bulk 3D material, taking palladium without lose of generality. In order to benchmark the robustness of our approach, we have performed classical MD simulations at temperatures from 50 K to 500 K every 50 K. In this case—to be able to perform lengthy MD reference simulations—we have used the embedded atom model (EAM) as the reference level of theory. We ran the simulations using ASE [**?**] coupled to an EAM potential [79**? ?**

? ? ?], with an integration step of 5.0 fs and 100,000 total steps for each temperature.

In Supplementary Fig. 5 we show the direct comparison between the radial distribution functions generated using the BIGDML model and the reference level of theory, both coupled to the MD interface of ASE and ran using the same settings. From this, we can see a perfect match between the machine learning generated trajectories and the reference simulations. The importance of this relies in the fact that, not only the harmonic regime is accurately described but also the anharmonic parts of the PES, results that confirm the effectiveness of our approach.

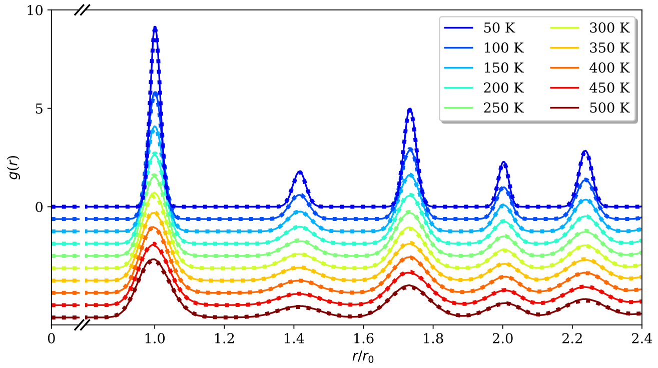

Supplementary Figure 5. Radial distribution function comparison between classical MD using EAM (continuous lines) and BIGDML (dotted lines) for different temperatures. The functions for each temperature are shifted downwards for clarity.

II. EXTENDED DESCRIPTION OF THE BENZENE/GRAPHENE SYSTEM

The Bz/graphene system has three minima that resemble those of the benzene dimer: the $\pi-\pi$ stacking structure (or parallel-displaced) as global minimum and the two local minima corresponding to parallel and T-shaped configurations, as displayed (see Fig. 7-B in the manuscript) [3]. The $\pi-\pi$ stacking configuration has an adsorption energy of -0.483 eV (≈-11 kcal mol^{-1}), and the parallel and T–shaped configurations have relative adsorption energies of 37 meV (0.85 kcal mol^{-1}) and 223 meV (5.14 kcal mol^{-1}), respectively, computed at the PBE+MBD level of theory [69, 70, 91]. The computed adsorption energy for the global minimum is in very good agreement with experimental measurements of 500±80 meV (11.5±1.8 kcal mol^{-1}) [92]. The different separation distances between the benzene molecule and the graphene layer for the different minima is reported in Fig. 7-B in the manuscript, where the separation of 3.30 Å in the case of the global minimum agrees with previously reported theoretical values [?].

SUPPLEMENTARY REFERENCES

[1] M. Veit, S. K. Jain, S. Bonakala, I. Rudra, D. Hohl, and G. Csányi, J. Chem. Theory Comput. **15**, 2574 (2019).
[2] B. Cheng, G. Mazzola, C. J. Pickard, and M. Ceriotti, Nature **585**, 217 (2020).
[3] H. E. Sauceda, V. Vassilev-Galindo, S. Chmiela, K.-R. Müller, and A. Tkatchenko, Nat. Commun. **12**, 442 (2021).
[4] V. L. Deringer, N. Bernstein, G. Csányi, C. Ben Mahmoud, M. Ceriotti, M. Wilson, D. A. Drabold, and S. R. Elliott, Nature **589**, 59 (2021).
[5] V. Ladygin, P. Korotaev, A. Yanilkin, and A. Shapeev, Comput. Mater. Sci. **172**, 109333 (2020).
[6] J. S. Smith, O. Isayev, and A. E. Roitberg, Chem. Sci. **8**, 3192 (2017).
[7] F. Noé, A. Tkatchenko, K.-R. Müller, and C. Clementi, Annu. Rev. Phys. Chem. **71**, 361 (2020).
[8] A. Tkatchenko, Nat. Commun. **11**, 4125 (2020).
[9] O. T. Unke, S. Chmiela, H. E. Sauceda, M. Gastegger, I. Poltavsky, K. T. Schütt, A. Tkatchenko, and K.-R. Müller, Chem. Rev. **121**, 1014210186 (2021).
[10] O. A. Von Lilienfeld, Angew. Chem. Int. Ed. **57**, 4164 (2018).
[11] K. T. Schütt, S. Chmiela, O. A. von Lilienfeld, A. Tkatchenko, K. Tsuda, and K.-R. Müller, *Machine Learning Meets Quantum Physics*, Vol. 968 (Springer Lecture Notes in Physics, 2020).
[12] F. Musil, A. Grisafi, A. P. Bartók, C. Ortner, G. Csányi, and M. Ceriotti, Chem. Rev. **121**, 9759 (2021).
[13] O. A. von Lilienfeld and K. Burke, Nat. Commun. **11**, 4895 (2020).
[14] J. A. Keith, V. Vassilev-Galindo, B. Cheng, S. Chmiela, M. Gastegger, K.-R. Müller, and A. Tkatchenko, Chem. Rev. **121**, 9816 (2021).
[15] W. Gao, S. P. Mahajan, J. Sulam, and J. J. Gray, Patterns **1**, 100142 (2020).

[16] F. Noé, G. De Fabritiis, and C. Clementi, Curr. Opin. Struc. Biol. **60**, 77 (2020).
[17] S. A. Ghasemi, A. Hofstetter, S. Saha, and S. Goedecker, Phys. Rev. B **92**, 045131 (2015).
[18] I. S. Novikov, K. Gubaev, E. V. Podryabinkin, and A. V. Shapeev, Mach. Learn.: Sci. Technol. **2**, 025002 (2021).
[19] N. Artrith, A. Urban, and G. Ceder, J. Chem. Phys. **148**, 241711 (2018).
[20] J. Byggmästar, K. Nordlund, and F. Djurabekova, Phys. Rev. Materials **4**, 093802 (2020).
[21] A. P. Bartók, J. Kermode, N. Bernstein, and G. Csányi, Phys. Rev. X **8**, 041048 (2018).
[22] A. P. Bartók, S. De, C. Poelking, N. Bernstein, J. R. Kermode, G. Csányi, and M. Ceriotti, Sci. Adv. **3**, e1701816 (2017).
[23] S. Chmiela, H. E. Sauceda, K.-R. Müller, and A. Tkatchenko, Nat. Commun. **9**, 3887 (2018).
[24] O. T. Unke and M. Meuwly, J. Chem. Theory Comput. **15**, 3678 (2019).
[25] C. Devereux, J. S. Smith, K. K. Davis, K. Barros, R. Zubatyuk, O. Isayev, and A. E. Roitberg, J. Chem. Theo. Comp. **16**, 4192 (2020).
[26] J. Behler, Int. J. Quantum Chem. **115**, 1032 (2015).
[27] K. T. Butler, D. W. Davies, H. Cartwright, O. Isayev, and A. Walsh, Nature **559**, 547 (2018).
[28] S. K. Wallace, A. van Roekeghem, A. S. Bochkarev, J. Carrasco, A. Shapeev, and N. Mingo, Phys. Rev. Research **3**, 013139 (2021).
[29] O. A. von Lilienfeld, K.-R. Müller, and A. Tkatchenko, Nat. Rev. Chem. **4**, 347 (2020).
[30] P. Seema, J. Behler, and D. Marx, Phys. Rev. Lett. **115**, 036102 (2015).
[31] K. T. Schütt, H. E. Sauceda, P.-J. Kindermans, A. Tkatchenko, and K.-R. Müller, J. Chem. Phys. **148**, 241722 (2018).
[32] V. L. Deringer, M. A. Caro, and G. Csányi, Nat. Commun. **11**, 5461 (2020).
[33] T. W. Ko, J. A. Finkler, S. Goedecker, and J. Behler, Nat. Commun. **12**, 398 (2021).
[34] O. T. Unke, S. Chmiela, M. Gastegger, K. T. Schütt, H. E. Sauceda, and K.-R. Müller, Nat. Commun. **12**, 7273 (2021).
[35] P. Rowe, V. L. Deringer, P. Gasparotto, G. Csányi, and A. Michaelides, J. Chem. Phys. **153**, 034702 (2020).
[36] P. Rowe, G. Csányi, D. Alfè, and A. Michaelides, Phys. Rev. B **97**, 054303 (2018).
[37] J. Behler, Angew. Chem. Int. Ed. **56**, 12828 (2017).
[38] N. Artrith and J. Behler, Phys. Rev. B **85**, 045439 (2012).
[39] G. H. Booth, A. Grüneis, G. Kresse, and A. Alavi, Nature **493**, 365 (2013).
[40] T. Gruber, K. Liao, T. Tsatsoulis, F. Hummel, and A. Grüneis, Phys. Rev. X **8**, 021043 (2018).
[41] A. Zen, J. G. Brandenburg, J. Klimeš, A. Tkatchenko, D. Alfè, and A. Michaelides, Proc. Natl. Acad. Sci. USA **115**, 1724 (2018).
[42] S. Chmiela, A. Tkatchenko, H. E. Sauceda, I. Poltavsky, K. T. Schütt, and K.-R. Müller, Sci. Adv. **3**, e1603015 (2017).
[43] S. Chmiela, H. E. Sauceda, I. Poltavsky, K.-R. Müller, and A. Tkatchenko, Comput. Phys. Commun. **240**, 38 (2019).
[44] G. Montavon, K. Hansen, S. Fazli, M. Rupp, F. Biegler, A. Ziehe, A. Tkatchenko, A. Lilienfeld, and K.-R. Müller, Advances in neural information processing systems **25**, 440 (2012).
[45] G. Montavon, M. Rupp, V. Gobre, A. Vazquez-Mayagoitia, K. Hansen, A. Tkatchenko, K.-R. Müller, and O. A. Von Lilienfeld, New J. Phys. **15**, 095003 (2013).
[46] F. Anselmi, L. Rosasco, and T. Poggio, Information and Inference: A Journal of the IMA **5**, 134 (2016).
[47] T. Poggio and F. Anselmi, *Visual cortex and deep networks: learning invariant representations* (MIT Press, 2016).
[48] M. Rupp, A. Tkatchenko, K.-R. Müller, and O. A. von Lilienfeld, Phys. Rev. Lett. **108**, 58301 (2012).
[49] H. E. Sauceda, S. Chmiela, I. Poltavsky, K.-R. Müller, and A. Tkatchenko, J. Chem. Phys. **150**, 114102 (2019).
[50] M. Hloucha and U. K. Deiters, Mol. Simul. **20**, 239 (1998).
[51] S. Chmiela, *Towards exact molecular dynamics simulations with invariant machine-learned models*, Doctoral thesis, Technische Universität Berlin, Berlin (2019).
[52] F. Faber, A. Lindmaa, O. A. von Lilienfeld, and R. Armiento, Int. J. Quantum Chem. **115**, 1094 (2015).
[53] M. J. Willatt, F. Musil, and M. Ceriotti, J.Chem. Phys. **150**, 154110 (2019).
[54] J. Behler and M. Parrinello, Phys. Rev. Lett. **98**, 146401 (2007).
[55] A. P. Bartók, R. Kondor, and G. Csányi, Phys. Rev. B **87**, 184115 (2013).
[56] H. Huo and M. Rupp, "Unified representation of molecules and crystals for machine learning," (2018), arXiv:1704.06439.
[57] K. T. Schütt, H. Glawe, F. Brockherde, A. Sanna, K. R. Müller, and E. K. U. Gross, Phys. Rev. B **89**, 205118 (2014).
[58] F. A. Faber, A. S. Christensen, B. Huang, and O. A. von Lilienfeld, J. Chem. Phys. **148**, 241717 (2018).
[59] Z. Li, J. R. Kermode, and A. De Vita, Phys. Rev. Lett. **114**, 096405 (2015).
[60] W. Pronobis, A. Tkatchenko, and K.-R. Müller, J. Chem. Theory Comput. **14**, 2991 (2018).
[61] J. Sólyom, *Fundamentals of the Physics of Solids: Volume I: Structure and Dynamics*, 1st ed. (Springer, 2008).
[62] I. Y. Zhang and A. Grüneis, Front. Mater. **6**, 123 (2019).
[63] D. Yoon, Y.-W. Son, and H. Cheong, Nano Lett. **11**, 3227 (2011).
[64] Y. Fan, Y. Xiang, and H. Shen, Nanotechnol. Rev. **8**, 415421 (2019).
[65] X.-F. Yang, A. Wang, B. Qiao, J. Li, J. Liu, and T. Zhang, Acc. Chem. Res. **46**, 1740 (2013).
[66] A. Wang, J. Li, and T. Zhang, Nat. Rev. Chem. **2**, 65 (2018).
[67] F. Doherty, H. Wang, M. Yang, and B. R. Goldsmith, Catal. Sci. Technol. **10**, 5772 (2020).
[68] A. Tkatchenko and M. Scheffler, Phys. Rev. Lett. **102**, 073005 (2009).
[69] A. Tkatchenko, R. A. DiStasio, R. Car, and M. Scheffler, Phys. Rev. Lett. **108**, 236402 (2012).
[70] A. Ambrosetti, A. M. Reilly, R. A. DiStasio, and A. Tkatchenko, J. Chem. Phys. **140**, 18A508 (2014).
[71] V. G. Ruiz, W. Liu, E. Zojer, M. Scheffler, and A. Tkatchenko, Phys. Rev. Lett. **108**, 146103 (2012).
[72] J. Hermann and A. Tkatchenko, Phys. Rev. Lett. **124**, 146401 (2020).
[73] F. Cleri and V. Rosato, Phys. Rev. B **48**, 22 (1993).

[74] M. S. Daw, S. M. Foiles, and M. I. Baskes, Mat. Sci. Eng. Rep. **9**, 251 (1993).
[75] H. E. Sauceda and I. L. Garzón, J. Phys. Chem. C **119**, 10876 (2015).
[76] J. George, G. Hautier, A. P. Bartók, G. Csányi, and V. L. Deringer, J. Chem. Phys. **153**, 044104 (2020).
[77] M. Lozada-Hidalgo, S. Hu, O. Marshall, A. Mishchenko, A. N. Grigorenko, R. A. W. Dryfe, B. Radha, I. V. Grigorieva, and A. K. Geim, Science **351**, 68 (2016).
[78] I. Poltavsky, L. Zheng, M. Mortazavi, and A. Tkatchenko, J. Chem. Phys. **148**, 204707 (2018).
[79] E. Tadmor, "EAM potential (LAMMPS cubic hermite tabulation) for Pd developed by Zhou, Johnson, and Wadley (2004); NIST retabulation v000," OpenKIM, `https://doi.org/10.25950/9edc9c7c` (2018).
[80] S. Gowtham, R. H. Scheicher, R. Ahuja, R. Pandey, and S. P. Karna, Phys. Rev. B **76**, 033401 (2007).
[81] N. Varghese, U. Mogera, A. Govindaraj, A. Das, P. K. Maiti, A. K. Sood, and C. N. R. Rao, Chem. Phys. Chem. **10**, 206 (2009).
[82] A. AlZahrani, Appl. Surf. Sci. **257**, 807 (2010).
[83] T. Gan and S. Hu, Microchim. Acta **175**, 1 (2011).
[84] B. D. Mohapatra, S. P. Mantry, N. Behera, B. Behera, S. Rath, and K. S. K. Varadwaj, Chem. Commun. **52**, 10385 (2016).
[85] A. Chakradhar, N. Sivapragasam, M. T. Nayakasinghe, and U. Burghaus, J. Vac. Sci. Technol. A **34**, 021402 (2016).
[86] S. Roychoudhury, C. Motta, and S. Sanvito, Phys. Rev. B **93**, 045130 (2016).
[87] M. Z. Tonel, I. V. Lara, I. Zanella, and S. B. Fagan, Phys. Chem. Chem. Phys. **19**, 27374 (2017).
[88] M. Z. Tonel, M. O. Martins, I. Zanella, R. B. Pontes, and S. B. Fagan, Comput. Theor. Chem. **1115**, 270 (2017).
[89] E. E. de Moraes, M. Z. Tonel, S. B. Fagan, and M. C. Barbosa, J. Mol. Model. **25**, 302 (2019).
[90] N. Ojaghlou, D. Bratko, M. Salanne, M. Shafiei, and A. Luzar, ACS Nano **14**, 7987 (2020).
[91] J. P. Perdew, K. Burke, and M. Ernzerhof, Phys. Rev. Lett. **77**, 3865 (1996).
[92] R. Zacharia, H. Ulbricht, and T. Hertel, Phys. Rev. B **69**, 155406 (2004).
[93] W. Fang, J. Chen, M. Rossi, Y. Feng, X.-Z. Li, and A. Michaelides, J. Phys. Chem. Lett. **7**, 2125 (2016).
[94] T. E. Markland and M. Ceriotti, Nat. Rev. Chem. **2**, 0109 (2018).
[95] M. Rossi, W. Fang, and A. Michaelides, J. Phys. Chem. Lett. **6**, 4233 (2015).
[96] P. Leinen, M. Esders, K. T. Schütt, C. Wagner, K.-R. Müller, and F. S. Tautz, Sci. Adv. **6**, eabb6987 (2020).
[97] A. Züttel, Materials Today **6**, 24 (2003).
[98] H. Kimizuka, S. Ogata, and M. Shiga, Phys. Rev. B **97**, 014102 (2018).
[99] D. E. Jiang and E. A. Carter, Phys. Rev. B **70**, 064102 (2004).
[100] X. W. Zhou, F. E. Gabaly, V. Stavila, and M. D. Allendorf, J. Phys. Chem. C **120**, 7500 (2016).
[101] J. Völkl, G. Wollenweber, K.-H. Klatt, and G. Alefeld, Z. Naturforsch. A **26**, 922 (1971).
[102] B. J. Heuser, D. R. Trinkle, N. Jalarvo, J. Serio, E. J. Schiavone, E. Mamontov, and M. Tyagi, Phys. Rev. Lett. **113**, 025504 (2014).
[103] G. L. Powell and J. R. Kirkpatrick, Phys. Rev. B **43**, 6968 (1991).
[104] A. P. Bartók, M. C. Payne, R. Kondor, and G. Csányi, Phys. Rev. Lett. **104**, 136403 (2010).
[105] W. Pronobis, *Towards more efficient and performant computations in quantum chemistry with machine learning*, Doctoral thesis, Technische Universität Berlin, Berlin (2020).
[106] D. P. Kovács, C. v. d. Oord, J. Kucera, A. E. Allen, D. J. Cole, C. Ortner, and G. Csányi, J. Chem. Theory Comput. **17**, 7696 (2021).
[107] M. L. Braun, J. M. Buhmann, and K.-R. Müller, J. Mach. Learn. Res. **9**, 1875 (2008).
[108] V. N. Vapnik, *The Nature of Statistical Learning Theory* (Springer, New York, NY, 1995).
[109] K.-R. Müller, S. Mika, G. Ratsch, K. Tsuda, and B. Schölkopf, IEEE Trans. Neural Netw. Learn. Syst. **12**, 181 (2001).
[110] B. Schölkopf and A. J. Smola, *Learning with kernels: support vector machines, regularization, optimization, and beyond* (MIT press, 2002).
[111] C. K. Williams and C. E. Rasmussen, *Gaussian processes for machine learning* (MIT press Cambridge, MA, 2006).
[112] N. Thomas, T. Smidt, S. Kearnes, L. Yang, L. Li, K. Kohlhoff, and P. Riley, "Tensor field networks: Rotation-and translation-equivariant neural networks for 3d point clouds," (2018), arXiv:1802.08219.
[113] K. Schütt, O. Unke, and M. Gastegger, in *Int. Conf. Mach. Learn.* (PMLR, 2021) pp. 9377–9388.
[114] Y. A. LeCun, L. Bottou, G. B. Orr, and K.-R. Müller, in *Neural networks: Tricks of the trade* (Springer, 2012) pp. 9–48.
[115] P. Giannozzi, S. Baroni, N. Bonini, M. Calandra, R. Car, C. Cavazzoni, D. Ceresoli, G. L. Chiarotti, M. Cococcioni, I. Dabo, A. D. Corso, S. de Gironcoli, S. Fabris, G. Fratesi, R. Gebauer, U. Gerstmann, C. Gougoussis, A. Kokalj, M. Lazzeri, L. Martin-Samos, N. Marzari, F. Mauri, R. Mazzarello, S. Paolini, A. Pasquarello, L. Paulatto, C. Sbraccia, S. Scandolo, G. Sclauzero, A. P. Seitsonen, A. Smogunov, P. Umari, and R. M. Wentzcovitch, J. Phys.: Condens. Matter **21**, 395502 (2009).
[116] P. Giannozzi, O. Andreussi, T. Brumme, O. Bunau, M. B. Nardelli, M. Calandra, R. Car, C. Cavazzoni, D. Ceresoli, M. Cococcioni, N. Colonna, I. Carnimeo, A. D. Corso, S. de Gironcoli, P. Delugas, R. A. DiStasio, A. Ferretti, A. Floris, G. Fratesi, G. Fugallo, R. Gebauer, U. Gerstmann, F. Giustino, T. Gorni, J. Jia, M. Kawamura, H.-Y. Ko, A. Kokalj, E. Kçkbenli, M. Lazzeri, M. Marsili, N. Marzari, F. Mauri, N. L. Nguyen, H.-V. Nguyen, A. O. de-la Roza, L. Paulatto, S. Poncé, D. Rocca, R. Sabatini, B. Santra, M. Schlipf, A. P. Seitsonen, A. Smogunov, I. Timrov, T. Thonhauser, P. Umari, N. Vast, X. Wu, and S. Baroni, J. Phys.: Condens. Matter **29**, 465901 (2017).
[117] V. Blum, R. Gehrke, F. Hanke, P. Havu, V. Havu, X. Ren, K. Reuter, and M. Scheffler, Comput. Phys. Commun. **180**, 2175 (2009).

[118] V. Kapil, M. Rossi, O. Marsalek, R. Petraglia, Y. Litman, T. Spura, B. Cheng, A. Cuzzocrea, R. H. Meiner, D. M. Wilkins, B. A. Helfrecht, P. Juda, S. P. Bienvenue, W. Fang, J. Kessler, I. Poltavsky, S. Vandenbrande, J. Wieme, C. Corminboeuf, T. D. Khne, D. E. Manolopoulos, T. E. Markland, J. O. Richardson, A. Tkatchenko, G. A. Tribello, V. Van Speybroeck, and M. Ceriotti, Comput. Phys. Commun. **236**, 214 (2019).

[119] A. Togo and I. Tanaka, Scr. Mater. **108**, 1 (2015).
