# Supplementary material for: BIGDML—Towards accurate quantum machine learning force fields for materials
Source: Nat Commun. 2022 Jun 29;13:3733. doi: 10.1038/s41467-022-31093-x (PMC9243122; doi:10.1038/s41467-022-31093-x)
Supplement: Supplementary file 3 — Description of Additional Supplementary Files [file 41467_2022_31093_MOESM3_ESM.docx]

Description of Additional Supplementary Files

Videos

File Name: Supplementary Video 1
Description: Animated version of Fig. 8B. This represents the hydrogen sampling of the O-site at 100K using CLMD.

File Name: Supplementary Video 2
Description: Animated version of Fig. 8B. This represents the hydrogen sampling of the O-site at 100K using PIMD.

File Name: Supplementary Video 3
Description: Animated version of Fig. 8B. This represents the hydrogen sampling of the O-site at 600K using CLMD.

File Name: Supplementary Video 4
Description: Animated version of Fig. 8B. This represents the hydrogen sampling of the O-site at 600K using PIMD.

Software

File Name: BIGDML_phonon_calculations.py
Description: Python script used for the phonon calculations.

File Name: BIGDML_MEB_calculations.py
Description: Python script used for minimum-energy path (MEB) calculations.
