# Supplementary figures and images for: BIGDML—Towards accurate quantum machine learning force fields for materials
Source: Nat Commun. 2022 Jun 29;13:3733. doi: 10.1038/s41467-022-31093-x (PMC9243122; doi:10.1038/s41467-022-31093-x)

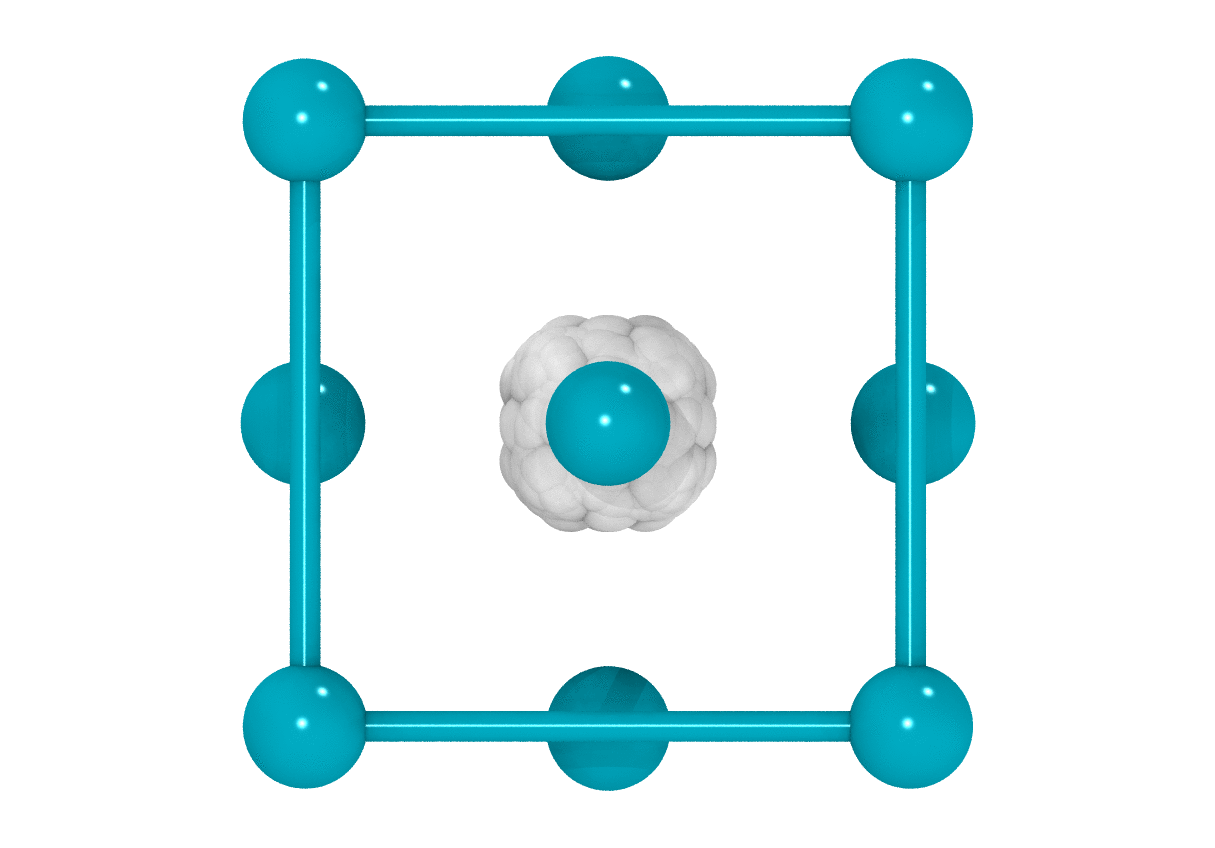

Supplement: Supplementary file 4 — Supplementary Video 1 [file 41467_2022_31093_MOESM4_ESM.gif]

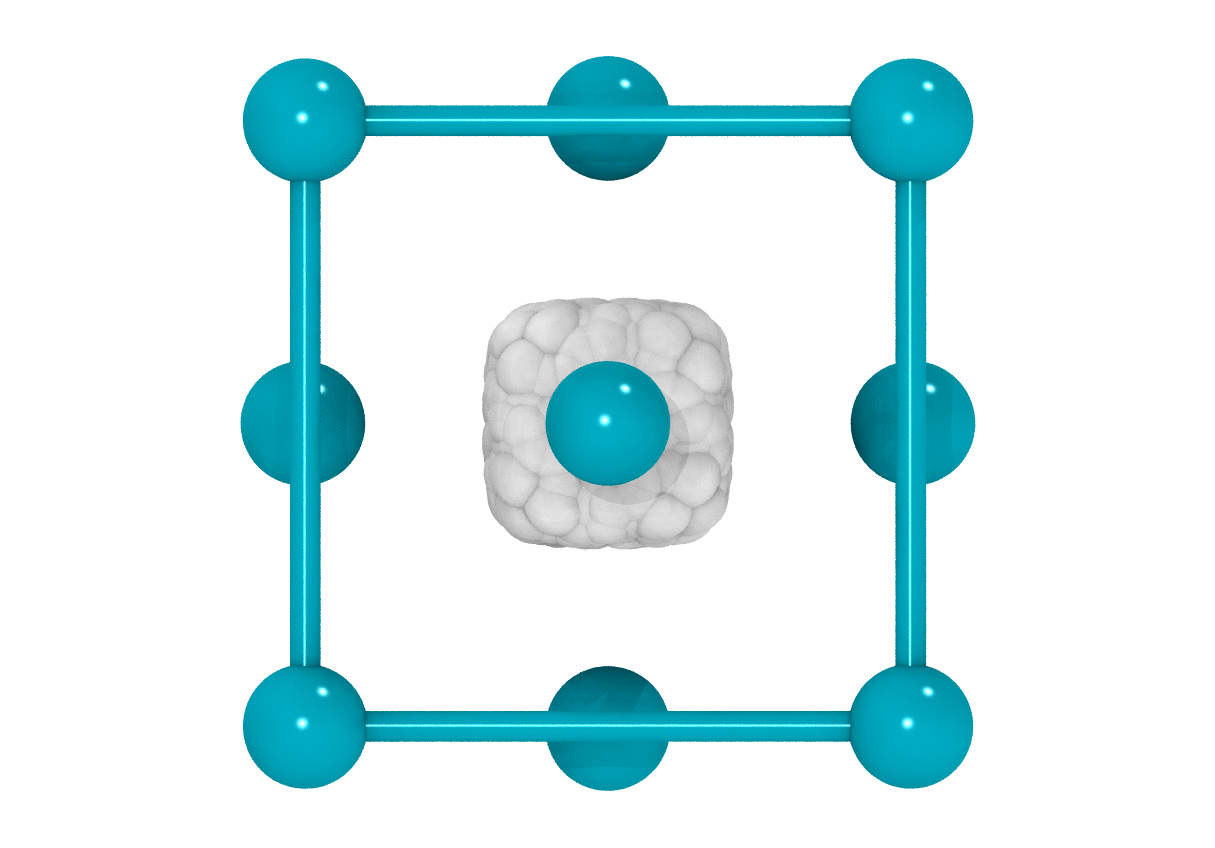

Supplement: Supplementary file 5 — Supplementary Video 2 [file 41467_2022_31093_MOESM5_ESM.gif]

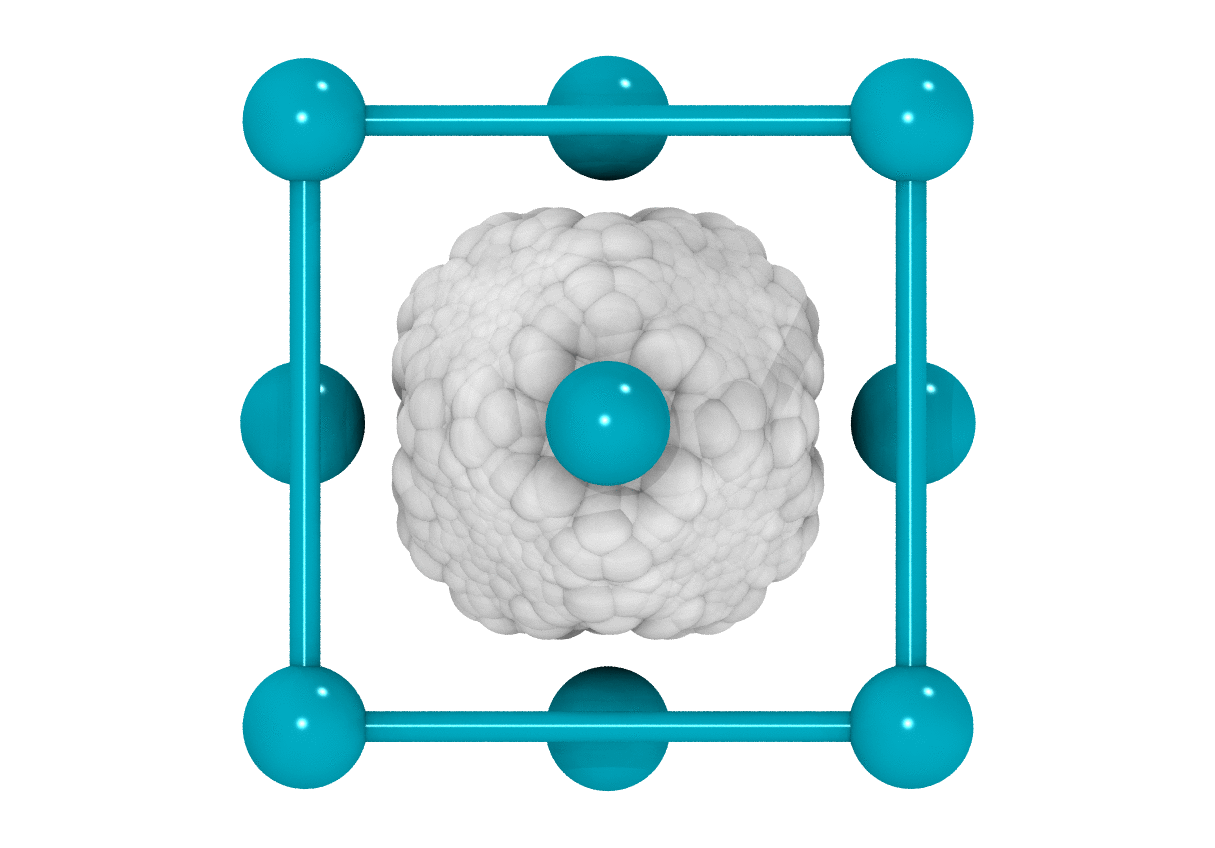

Supplement: Supplementary file 6 — Supplementary Video 3 [file 41467_2022_31093_MOESM6_ESM.gif]

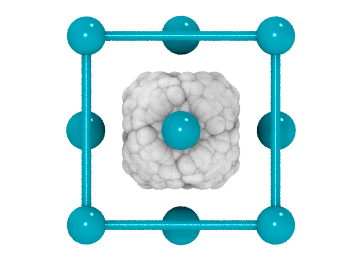

Supplement: Supplementary file 7 — Supplementary Video 4 [file 41467_2022_31093_MOESM7_ESM.gif]
